# So, you want to create a frog cell line? A guide to establishing frog skin cell lines from tissue explants

**DOI:** 10.1016/j.mex.2022.101693

**Published:** 2022-04-08

**Authors:** Maxwell P. Bui-Marinos, Lauren A. Todd, Alexander J. Douglas, Barbara A. Katzenback

**Affiliations:** Department of Biology, University of Waterloo, Waterloo, Ontario, N2L3G1, Canada

**Keywords:** Amphibian, Xenopus laevis, Rana sylvatica, Tissue culture explant, Skin, Primary cell culture, Continuous cell cultures, Cell line, In vitro systems

## Abstract

Skin is an important interface with the external environment and investigating amphibian skin cell biology will improve our understanding of how environmental factors such as pathogens and pollutants are contributing to global amphibian declines. There is a critical need for *in vitro* systems to facilitate conservation research in model and non-model amphibians and the creation of new amphibian cell lines will play a significant role in reducing or even replacing the use of live animals for *in vivo* studies by providing an *in vitro* alternative. Here, we detail an adapted protocol for the generation of spontaneously arising cell lines from frog skin tissues, without the need for immortalization steps. Expanding the amphibian invitrome will foster and expedite new research in amphibian gene function, cellular responses, host-pathogen interactions, and toxicology. The following customizations to traditional tissue explant generation procedures have facilitated the successful generation of adherent skin epithelial-like cell lines from *Xenopus laevis* and can be further adapted for use with different frog species, such as *Rana sylvatica*, and different tissues:

• Osmotic adjustment of culture medium and solutions for different amphibian species.
• Use of small tissue explants, instead of enzymatic digestion of tissues, and gentle spotting of these tissue explants onto the growth surface of tissue culture flasks to promote better tissue adherence.
• Partial replacement of medium to allow accumulation of potential endogenous growth factors in cultures.

## Graphical abstract

**Figure:**
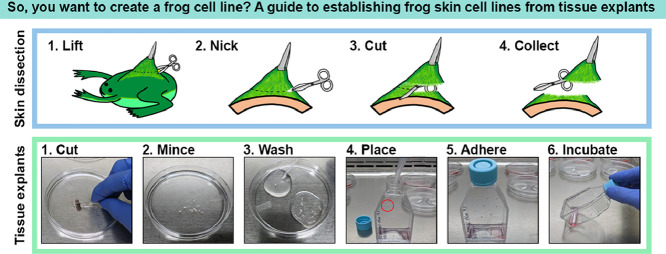

## Specifications table

**Table:** 

Subject Area:	Agricultural and Biological Sciences
More specific subject area:	Animal cell culture
Method name:	Tissue explant method for frog skin cell line generation
Name and reference of original method:	R.I. Freshney. Culture of animal cells: A manual of basic technique (5^th^ ed), Wiley-Blackwell (2005) p.696. https://doi.org/10.1002/9780471747598 M.P. Bui-Marinos, J.F.A. Varga, N.T.K. Vo, N.C. Bols, B.A. Katzenback. Xela DS2 and Xela VS2: Two novel skin epithelial-like cell lines from adult African clawed frog (*Xenopus laevis*) and their response to an extracellular viral dsRNA analogue, Dev. Comp. Immunol. 112 (2020) 103759. https://doi.org/10.1016/j.dci.2020.103759
Resource availability:	N/A

## Materials

• Gloves
• Lab coat
• Cell culture-grade sterile water (Lonza, VWR, Cat. No. CA12001-356; or ultrapure water from Milli-Q Reference system)
• Phosphate-buffered saline (PBS; Lonza, Fisher Scientific, Cat. No. BW17-517Q; or prepared in-house)
• Leibovitz's L-15 medium (HyClone, Fisher Scientific, Cat. No. SH3052501; or Wisent, Cat. No. 223-050-XK)
• Fetal bovine serum (FBS; HyClone, Fisher Scientific, Cat. No. SH3039603; or Avantor Seradigm Premium Grade FBS, VWR, Cat. No. 89510-194)
• 100 × penicillin/streptomycin solution (HyClone, Fisher Scientific, Cat. No. SV30010), 10,000 U/mL penicillin and 10,000 µg/mL streptomycin stock solution
• Gentamicin sulfate (Fisher Bioreagent, Fisher Scientific, Cat. No. BP918-1), 12.5 mg/mL stock solution
• Amphotericin B (Gibco, Fisher Scientific, Cat. No. 15290026), 250 µg/mL stock solution
• Trypsin-EDTA (Wisent, Cat. No. 325-043-CL)
• β-mercaptoethanol (Sigma-Aldrich, Cat. No. M6250)
• Biosafety cabinet
• Spray bottle containing 70% ethanol
• Beaker containing 70% ethanol
• 100 mm × 15 mm petri dishes, sterile (Fisher Scientific, Cat. No. FB0875713)
• *Xenopus laevis* frog(s)
• Beaker for euthanasia, 1000 mL for *Xenopus* sp., 500 mL for smaller frog species
• Ethyl 4-aminobenzoate (Benzocaine; Fisher Scientific, Cat. No. AC150781000)
• Absolute ethanol
• Scalpel handle with attached scalpel blade (#10)
• Paper towel
• Forceps
• Dissection scissors
• Polypropylene transfer pipette, sterile (VWR, Cat. No. 414004-003)
• 25 cm^2^ plug-seal tissue culture treated flask (BioLite, Thermo Fisher Scientific, Cat. No. 12-556-012)
• 75 cm^2^ plug-seal tissue culture treated flask (BioLite, Thermo Fisher Scientific, Cat. No. 12-556-013)
• 1 mL pipette
• 1 mL pipette tips
• 5 mL serological pipettes
• 10 mL serological pipettes
• Pipette aid
• Incubator (without added CO_2_) capable of maintaining 26°C
• Inverted phase-contrast microscope (optional: equipped with a camera)
• Autoclavable waste bags
• Sterile 0.22 µm PES syringe filter (FroggaBio, Cat. No. SF0.22PES)
• Sterile 20 mL syringe (BD, Fisher Scientific, Cat. No. 22-124-967)
• 15 mL and 50 mL conical tubes
• Centrifuge
• Dimethyl sulfoxide (Fisher Scientific, Cat. No. BP231-1)
• Cooler and ice
• P1000 micropipette and sterile tips
• Cryopreservation tubes (Nunc, Thermo Fisher Scientific, Cat. No. 375418)
• Cell freezing container (e.g., Mr. Frosty, Thermo Fisher Scientific, Cat. No. 5100-0001)
• Liquid nitrogen storage tank
• Cryopreservation storage boxes
• Hemocytometer
• 0.4% Trypan blue dye (Lonza, VWR, Cat. No. 17-942E)
• -80°C Freezer
• Insulated cryogenic gloves
• Heated water bath

## Generation of frog skin cell line protocol

As the terminology which *in vitro* biologists use to describe cell cultures can be inconsistent, vague, or redundant, the following terms have been defined for clarity. The term “primary cell cultures” is used to refer to cells prior to the first subculturing event. The term “continuous cell cultures”, roughly synonymous with the terms “established cell cultures” and “indefinite cell cultures”, is used to refer to a culture which has been propagated *ex vivo* over a long term (>15-20 subculturing events) and does not display evidence of senescence or a reduced capacity for propagation. In the stricter sense, the term “cell line” refers to a cell culture derived from a single cell that is propagated to give rise to monotypic progeny. In the more general sense, the term “cell line” can refer to non-primary cultured cells. In this method article, we have chosen to use the general meaning of the term “cell line” to refer to the continuous frog skin cell lines we have successfully generated. If a researcher wishes to establish a “cell line” from a single cell, the below method could be used and once a continuous cell culture was established, limiting-dilution or single cell sorting can be performed to initiate monotypic cell lines in which the progeny arose from a single cell.

**IMPORTANT:** Unless otherwise stated, all the steps below should be performed in a biosafety cabinet and equipment/containers should be sprayed or wiped with 70% ethanol to ensure sterility.

### Preparation of media and cell culture reagents

To generate frog skin cell lines, a variety of sterile solutions and media are required. Since amphibian cells can differ in osmolality, it is important to adjust commercially available media (e.g. Leibovitz's L-15 medium is typically 300 – 340 mOsmol/kg) typically used for human, mouse, and fish cell lines to be suitable for use with amphibian cells. For example, the mean osmolality of adult *Xenopus* serum is 245 mOsmol/kg [1] and *Rana sylvatica* plasma ranges from 187 – 419 mOsmol/kg in the spring and winter months, respectively [2]. To establish cell cultures, we opted to use a higher concentration of fetal bovine serum (FBS) to provide ample growth factors to the tissue explants. Once the cell lines were established, we performed optimization experiments (outlined in “*Optimization of culture conditions for frog cell lines”* section below and described in [3]) to determine a concentration of FBS that promoted optimal cell growth while lessening the requirement of FBS in consideration of animal ethics and reagent costs. In this section, we report the use of amphibian-adjusted solutions for use with cells derived from *Xenopus laevis* and *Rana sylvatica*.

• PBS stock (1×): 137 mM NaCl, 2.7 mM KCl, 10 mM Na_2_HPO_4_, 1.8 mm KH_2_PO_4_ in sterile cell culture water, pH to 7.4 using HCl. For example, to prepare a 1 L solution, add 8 g NaCl, 0.2 g KCl, 1.44 g Na_2_HPO_4_, 0.24 g KH_2_PO_4_ to 800 mL of cell culture water, perform pH adjustments, and then top the solution to 1 L with cell culture water. Filter sterilize this solution using a 0.22 µm PES filter.
• Amphibian 70% Leibovitz's L-15 Medium (A_70_L-15): Sterile Leibovitz's L-15 medium diluted to 70% by mixing seven parts of L-15 medium with three parts sterile cell culture water to adjust for amphibian osmolarity.
• Xela establishment medium (passages < 4): A_70_L-15 medium further supplemented with an additional 20% FBS, 200 U/mL penicillin, 200 μg/mL streptomycin, 50 μg/mL gentamicin sulfate, and 1 μg/mL amphotericin B. For example, combine 100 mL of A_70_L-15, 20 mL of FBS, 2.5 mL of 100 × penicillin/streptomycin solution, 0.5 mL of 12.5 mg/mL gentamicin sulfate stock solution, 0.5 mL of 250 µg/mL amphotericin B stock solution.
• Xela early passage medium (passages ∼ 4 - 15): A_70_L-15 medium further supplemented with an additional 15% FBS, 100 U/mL penicillin, 100 μg/mL streptomycin, and 50 μg/mL gentamicin sulfate.
• Xela complete medium (passages > 15 - 20): A_70_L-15 medium further supplemented with an additional 15% FBS (without antibiotics).
• Amphibian 70% phosphate-buffered saline (A_70_PBS): Dilute PBS to 70% of the original salt concentration by mixing seven parts of PBS with three parts sterile cell culture water.
• Amphibian 70% phosphate-buffered saline with antibiotics (A_70_PBS-A): A_70_PBS supplemented with 200 U/mL penicillin, 200 μg/mL streptomycin, 50 μg/mL gentamicin sulfate, and 1 μg/mL amphotericin B.
• 70% trypsin solution: Dilute Trypsin-EDTA solution with sterile cell culture water to 70% of the original concentration by mixing seven parts trypsin with three parts sterile cell culture water.
• **Note:** We have adapted this protocol for use in other frog species such as *R. sylvatica*. In doing so, we have modified the reagents described above. We have been successful in establishing cell lines from *R. sylvatica* skin using the media formulations outlined below.
• Amphibian 60% Leibovitz's L-15 medium (A_60_L-15): Sterile Leibovitz's L-15 medium diluted to 60% with cell culture water by mixing six parts of L-15 medium with four parts sterile cell culture water.
• Rasy establishment medium: A_60_L-15 medium further supplemented with an additional 20% FBS, 200 U/mL penicillin, 200 μg/mL streptomycin, 50 μg/mL gentamicin sulfate, 2.5 μg/mL amphotericin B, and 3.5 µL/L β-mercaptoethanol. For example, combine 100 mL A_60_L-15 medium, 20 mL FBS, 2.5 mL 100 × penicillin/streptomycin solution, 0.5 mL 12.5 mg/mL gentamicin sulfate stock solution, 1.25 mL 250 µg/mL amphotericin B stock solution, and 0.35 µL β-mercaptoethanol.
• Rasy early passage medium (up to passage 10): A_60_L-15 medium further supplemented with an additional 20% FBS, 100 U/mL penicillin, 100 μg/mL streptomycin, and 50 μg/mL gentamicin sulfate, 3.5 µL/L β-mercaptoethanol, and 20% cell conditioned medium which had been collected from Xela DS2 passages of ≥ 90% confluency.
• Rasy complete medium (passages > 10): A_60_L-15 medium further supplemented with an additional 20% FBS and 20% cell-conditioned medium collected from previous *R. sylvatica* cell lines of the same identity.
• Amphibian 60% phosphate-buffered saline (A_60_PBS): Dilute PBS to 60% of the original salt concentration using cell culture water by mixing 6 parts of PBS with 4 parts of sterile cell culture water.
• Amphibian 60% phosphate-buffered saline with antibiotics (A_60_PBS-A): A_60_PBS supplemented with 200 U/mL penicillin, 200 μg/mL streptomycin, 50 μg/mL gentamicin sulfate, and 2.5 μg/mL amphotericin B.
• 60% trypsin solution: Dilute Trypsin-EDTA solution with sterile cell culture water to 60% of the original concentration by mixing 6 parts trypsin with 4 parts sterile cell culture water.

### Frog dissection and tissue explant cultures

1. All reagents, consumables, and dissection equipment should be sterile and handled in a biosafety cabinet. Dissection equipment should be placed in a beaker containing 70% ethanol to sterilize tools. Ensure the 70% ethanol solution covers the portion of the tools that will contact the animal during dissection. Dissection tools should be allowed to air dry in the biosafety cabinet prior to dissection. Alternatively, tools may be autoclaved prior to use.
2. In advance of frog euthanasia, prepare labeled petri dishes containing sterile APBS + antibiotics (A_60_PBS-A for *R. sylvatica*, A_70_PBS-A for *X. laevis*). At least four petri dishes containing APBS-A should be prepared per tissue being dissected.
3. Prepare benzocaine solution (final concentration 300 mg/L) for frog euthanasia by overdose. **IMPORTANT:** Dissolve the benzocaine in a small volume of ethanol first (should not exceed 2% of total volume of water the frog is placed in for euthanasia), then slowly add the benzocaine solution to the water while mixing to prevent benzocaine from precipitating out of solution. To avoid temperature shock, ensure the water is at the housing temperature of the animal. Be aware that addition of benzocaine to cold water may cause precipitation of benzocaine.
4. Place the frog in the benzocaine solution and watch for cessation of respiration and heartbeat. **IMPORTANT:** Confirm euthanasia by lightly pinching the frog at the feet and monitoring for any nerve response. If there is a response (i.e., muscle contraction), allow the frog to sit in the benzocaine solution for a longer period. Once there is no response, use a fresh scalpel blade to perform cervical severance. Always refer to current animal care guidelines for acceptable euthanasia methods that prevent or minimize pain to the animal.
5. Transfer the frog to a sterile biosafety cabinet. Use a paper towel to remove excess liquid from the frog before placing it on a new paper towel and spraying the animal with 70% ethanol.
6. Dissect desired tissues from euthanized *X. laevis* using sterile instruments in the biosafety cabinet. Dissect dorsal skin tissue by pinching the posterior portion of the dorsal skin with forceps and nick the skin tissue to create an opening (Fig. 1A). Using the forceps to hold up this tissue where the nick was made, insert the scissors into the opening in the skin and cut along each of the sides of the dorsal skin to the anterior part of the dorsal skin tissue (Fig. 1B) to excise an oval piece of skin.

**Fig. 1:**
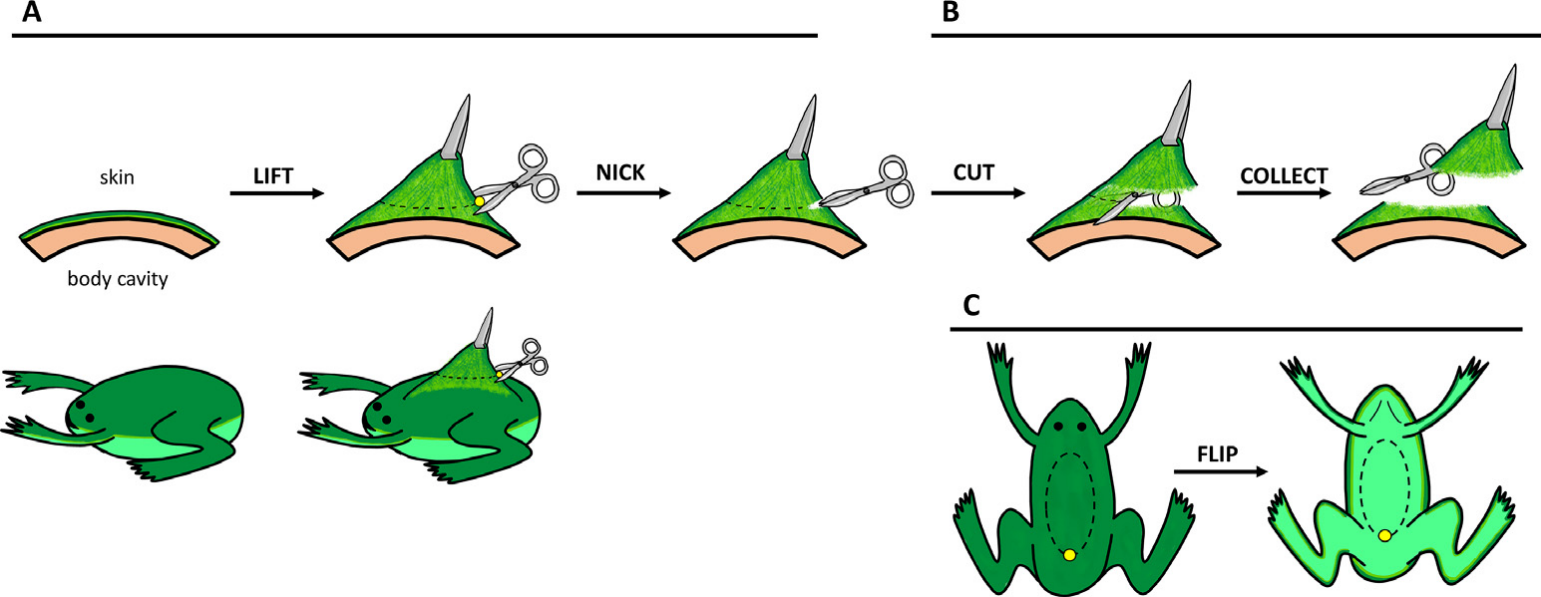
**The tissue dissection procedure.** (**A**) The posterior end of dorsal or ventral skin is gently lifted using a pair of forceps to separate the skin from underlying tissue. The skin needs to be lifted only a small amount and the forceps should grip the skin on the posterior end of the desired skin tissue just above the nicking point (yellow dot) to minimize tissue damage. Note: the diagram exaggerates the position of the forceps relative to the initial nick in the skin and the amount of lift of the skin for illustrative purposes. (**B**) Holding the piece of skin tissue at the posterior end where the nick was made with the scissors, carefully cut along each side of the skin (dashed line), alternating between each side, to separate and collect the skin tissue from the frog. (**C**) Depictions of the dorsal (left) and ventral (right) regions of *Xenopus laevis* targeted for skin dissection. The yellow dot indicates the point to nick with the scissors and then hold with the forceps, while the dashed line indicates the oval skin tissue section to be excised.
7. Immediately place the excised tissue into the first pre-prepared, labeled petri dish containing APBS-A.
8. To collect ventral skin, flip the frog over and spray it again with 70% ethanol before repeating the process (outlined in Steps 6 and 7 of this Section) with the ventral skin (Fig. 1C). **IMPORTANT**: When lifting and cutting the ventral skin, ensure not to cut into the peritoneal cavity and/or intestinal tract.
9. Use a scalpel blade to cut away the edges of the tissue to remove portions that may extend down the side of the frog and retain the central portion that corresponds to dorsal or ventral skin. From this central piece of skin tissue (Fig. 2A), cut 10 – 15 pieces that are approximately 3 mm x 3 mm in size, or however many pieces are possible given the size of the excised tissue. Transfer these pieces of tissue to the second petri dish of the series containing fresh APBS-A using a sterile transfer pipette (Fig. 2B). **IMPORTANT:** Minimize the transfer of APBS-A from the first petri dish to the second petri dish. **NOTE**: Tissue pieces may get stuck inside the walls of the transfer pipette but avoid attempting to purposely flush it out with vigorous pipetting. The incidence of tissue pieces getting stuck to the inside walls of the transfer pipette can be reduced by pre-wetting the inside of the transfer pipette with APBS-A.

**Fig. 2:**
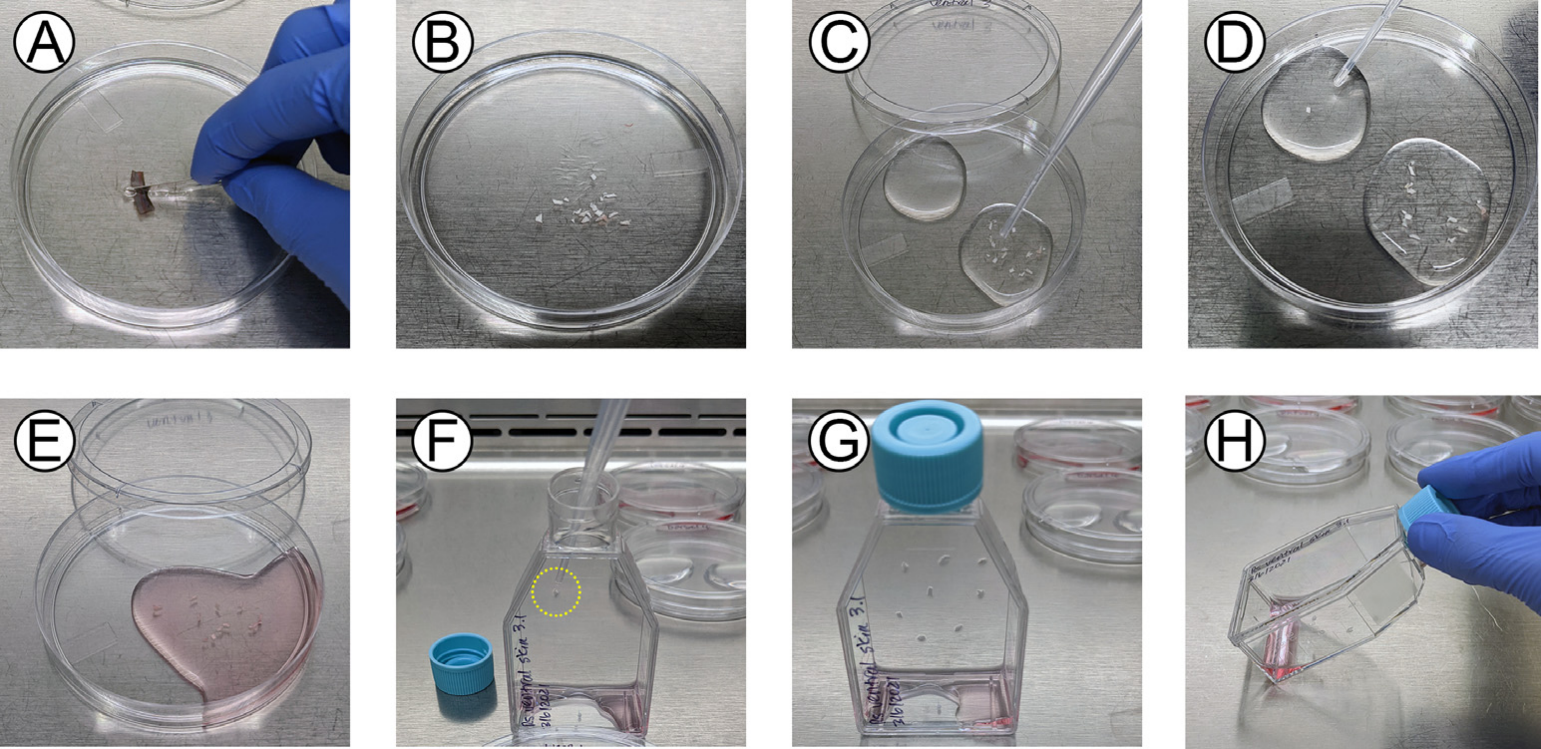
**The tissue explant procedure.** (**A**) Cutting of larger dissected tissue pieces into smaller pieces. (**B**) Transfer of the smaller tissue pieces into APBS-A. (**C**) First wash of the tissue pieces in APBS-A. (**D**) Second wash of the tissue pieces in APBS-A. (**E**) Transfer of the tissue pieces to establishment medium. (**F**) Placement of individual tissue pieces on the bottom (vertical surface when standing) of a 25 cm^2^ flask. (**G**) Adherence of the tissue pieces to the flask. (**H**) Addition of fresh establishment medium to the bottom of the flask and transfer of the flask to its standard position.
10. Use a sterile transfer pipette to wash the tissue pieces in APBS-A by gently pipetting the pieces up and down in the solution (Fig. 2C).
11. Transfer the small tissue pieces to the third petri dish containing fresh APBS-A and repeat the washing procedure described above (Fig. 2D). **IMPORTANT:** Minimize the transfer of APBS-A from the second petri dish to the third petri dish. **NOTE:** Two separate pools of APBS-A can be placed in a single petri dish (as shown in Fig. 2D) if a small number of tissue pieces (10 or less) are anticipated and the user can ensure the pools of ABPS-A do not merge. Otherwise, it is best to have separate petri dishes filled with APBS-A.
12. Transfer the small tissue pieces to a fourth petri dish containing fresh APBS-A or establishment medium (Fig. 2E). Again, try to minimize the amount of solution transferred from the third APBS-A wash. Rinse the tissues in the fresh APBS-A with a new sterile transfer pipette. **NOTE**: This final wash can be performed in establishment medium if desired (as pictured in Fig. 2E), as this may help to maintain the viability of the tissue pieces if several tissue explant cultures are being prepared simultaneously.
13. Hold a 25 cm^2^ plug-seal tissue culture treated flask upright and carefully transfer 4-6 tissue pieces into the flask using a sterile transfer pipette, gently placing them on the vertical surface corresponding to the bottom of the flask (Fig. 2F). Place the tissue pieces in as many flasks as possible, ensuring not to surpass 6 tissue pieces in one flask. Try to transfer as little of the wash solution as possible, but any excess will be pooled at the bottom of the flask which can be collected and removed. Try to space the tissue pieces far apart so that migrating/proliferating cells from one tissue piece will have plenty of room and not be restricted by potential contact inhibition (with other cells or the edges of the tissue culture vessel).
14. Leave the flask standing upright with the cap on for 5 – 10 minutes (Fig. 2G); we find that this helps with the attachment of the tissue piece to the bottom of the flask. **NOTE**: While tissue pieces are protected by residual APBS-A (or establishment medium) surrounding them, leaving the tissue pieces exposed to air for longer than 10 minutes is not recommended. It is important to always monitor the tissue pieces during this 5-10 min period to ensure they do not dry. Perform Steps 13 and 14 for each flask if there are sufficient tissue pieces to set up multiple flasks.
15. While the flask is still upright, carefully add 1.5 mL of establishment medium such that it drops to the bottom of the flask, being careful not to disrupt the tissue pieces.
16. Very gently tilt the flask from the upright position into the standard position (Fig. 2H). This will allow the fresh establishment medium to cover the tissue pieces. **NOTE**: In some cases, very gentle back-and-forth tilting of the flask is needed to help spread the establishment medium over the bottom of the flask. It is important to move very slowly, as even mild agitation can cause the tissue pieces to detach. Alternatively, we have found it beneficial to “pre-coat” the bottom of the tissue culture vessel with the desired medium prior to seeding the tissue pieces and titling the flask to the standing position (as described in Step 13 of this section). This helps to minimize the amount of gentle rocking or agitation needed to distribute the medium across the surface of the tissue culture vessel.
17. Successful tissue attachment varies on a case-by-case basis. The following describes what to do under different circumstances:
  a. If all pieces have attached, continue to Step 18 of this section.
  b. If most pieces (≥ 4/6 pieces) have attached, carefully remove floating pieces and place into a petri dish containing APBS-A or establishment medium. Repeat Steps 13 – 16 with any collected tissue pieces which did not attach to the flask. Any tissue pieces which remain unattached after the second attempt can be discarded. Continue to Step 18.
  c. If little or no pieces (≤ 2/6 pieces) have attached, use the same flask to repeat Steps 13 – 16. Use a sterile transfer pipette to reposition the tissue pieces. Any tissue pieces which remain unattached after the second attempt can be discarded. Continue to Step 18.
18. Very carefully place the flasks containing adhered tissue explants in an incubator set at the desired temperature (i.e., the thermal optimal temperature of the amphibian species being used to generate tissue explant cultures) in the absence of additional CO_2_. The tissue explant cultures will remain at this temperature throughout the development and maintenance of the cell line. **NOTE**: We have successfully initiated tissue culture explants for *X. laevis* at 26 °C and for *R. sylvatica* at 18 °C.
19. The next day, carefully add 1.5 mL of establishment medium to each flask (final volume of 3 mL per flask) and view the tissue explant pieces under a phase contrast microscope to establish a baseline for how the edges of the tissue pieces appear. Do not forget to take pictures!
20. It is very important to check on the explants daily in the first month – microbes grow quickly, thus contaminated flasks should be removed and put in an autoclave bag for destruction. It is best not to open the flask or try to save the other tissue pieces since it will be too late to save the tissue explant culture in most cases. However, if samples are precious, it may be necessary to open the contaminated flask in the biosafety cabinet and attempt to remove the contaminated tissue pieces/medium as needed. Here are our recommendations for trying to recover tissue explants from different types of contamination:
  a. Bacterial or single cell yeast contamination – remove the medium and wash with 4 – 5 mL of APBS-A multiple times. While washing, tilt the flask around such that the APBS-A solution contacts the entire growth surface of the flask. Replace with fresh establishment medium and do this for two more days. If contamination persists, repeat this process one more time. If there are still signs of contamination, treat the flask as biowaste and discard by autoclaving. If successful in removing signs of contamination, continue to Step 21.
  b. Hyphal/filamentous fungi contamination – fungal morphology will differ depending on the species, but this type of contamination often manifests as isolated fungal colonies either floating in the medium or growing out from a tissue piece. Remove the medium, any fungal colonies, and any tissue pieces attached to fungal colonies. Perform the wash regimen described in Step 20a. If successful in removing signs of contamination, continue to Step 21.
  c. Parasitic contamination – Morphology varies greatly depending on the nature of the parasite (e.g. protozoan, helminth), but they are typically clearly distinct from the cultured cells as large (sometimes macroscopic) and/or highly motile bodies. While we have not experienced issues with parasite contamination in skin tissue explants, they have been observed in explants from other tissue types such as extracellular trypanosomes in the blood, worms in lung tissue, or nodules in/on internal tissues. Parasitic contamination appears less detrimental to the tissue explant cultures as the media conditions used herein did not support long-term survival of the parasites. It is often sufficient to remove the medium which contains the parasite(s), wash the flask with APBS-A multiple times, and add fresh establishment medium. It is not necessary to do an intense wash regimen with parasitic contamination. Continue to Step 21.
21. In the first three weeks after establishing the tissue culture explant, change the explant culture medium twice a week using establishment medium. If the tissue explants remain well adhered and cells are migrating out of the tissue after 1 – 2 weeks, begin replenishing only half of the medium (from 3 mL in the flask, remove 1.5 mL and add in 1.5 mL of fresh establishment medium) twice a week for an additional 3 weeks. By leaving a portion of the original medium behind, beneficial endogenous compounds potentially secreted by the tissue and/or cells may concentrate and promote cellular outgrowth. If there is not yet significant outgrowth of the tissue explant cultures after 4 weeks of initiation, begin replenishing half of the medium every 7 - 14 days using early passage medium.
22. Throughout the first 3 weeks, regularly monitor the tissue explants for cell outgrowth (Fig. 3) using a phase contrast microscope. Record observations and take pictures of any new developments. Although dependent on frog species, tissue type, and growth temperature, initial cell migration from the tissue can typically be observed within the first week (Fig. 3A), and further outgrowth spreading along the surface of the flask can be observed with additional incubation (Fig. 3B). However, in some cases, initial cell migration from the tissue explant took upwards of 1 – 2 months.

**Fig. 3:**
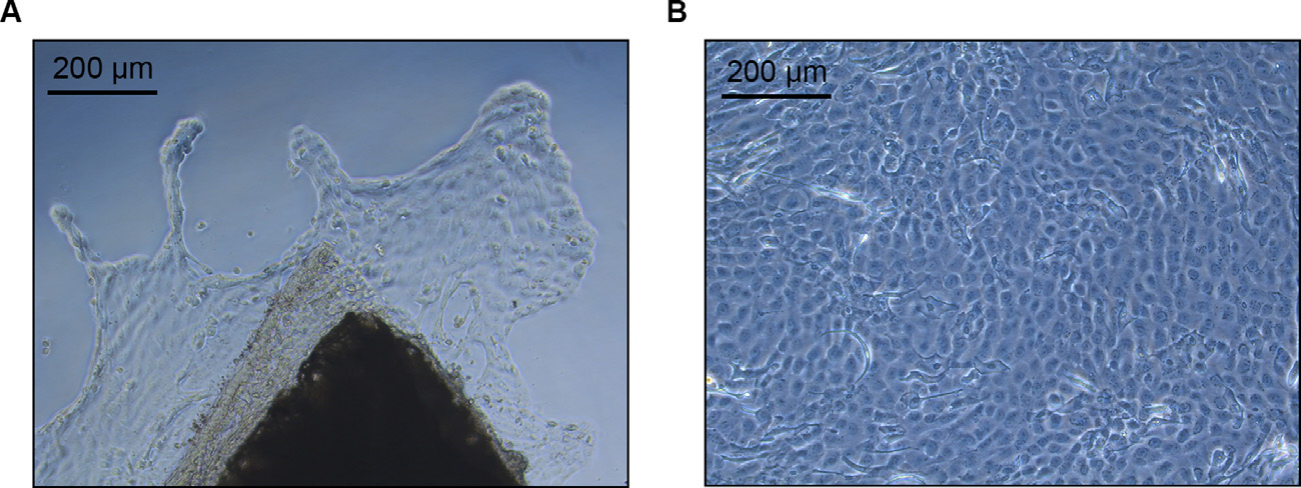
**Examples of cell outgrowth from frog skin tissue explants.** An example of (**A**) early cell outgrowth (day 2 post-explant procedure) and (**B)** significant cell outgrowth (day 8 post-explant procedure) from frog ventral skin tissue explants. Scale bars = 200 µm. Images of *R. sylvatica* ventral skin explants are provided for illustrative purposes, however similar observations were made for *X. laevis* skin tissue explants.
23. Determining when to first try splitting the tissue explant cell culture is challenging. This decision is usually based on observations of cell outgrowth over the past few days. Usually, we have waited until approximately 50% of the growth surface in the tissue culture vessel is covered with cells and we have observed signs of cell division. However, if approximately 50% confluency is not observed and the observed rapid outgrowth is now slowing down with tightly packed cells in the center, even though there is space surrounding the cell islands, this is likely the right time to split the cells. Similarly, we have also observed that some cells will start losing adherence due to contact inhibition (e.g., abutting the edge of the flask or another island of cell outgrowth). In these cases, we may dissociate the cells but place them back in the same flask with the original tissue explants if we suspect there are too few cells to be seeded into a new flask. In these cases, we only do a brief trypsin treatment to detach the cells, but not the tissue explant, from the tissue culture flask. Thus, it is important to carefully monitor the tissue explants and cell outgrowth daily. In our experience, we have found that initial cell migration and proliferation can take 1 – 2 months or sometimes longer depending on the tissue, growth conditions, and overall general proliferative capacity of the cells before the primary cell culture can first be subcultured.
24. Once tissue explant cultures exhibit significant cell growth characterized by ∼50% or more of the growth surface being covered by cells, collect adherent cells by removing the medium and washing 2 – 3 times with APBS-A prior to the addition of 0.5 mL of amphibian trypsin solution (70% for *X. laevis* and 60% for *R. sylvatica*).
25. Monitor cell detachment under a phase contrast microscope. We recommend avoiding tapping the flask to expedite cell detachment, as it can cause the tissue pieces to detach.
26. Upon cell detachment, carefully add 0.5 mL of early passage medium to inactivate the trypsin. Transfer the detached cells into a fresh 25 cm^2^ plug-seal tissue culture flask, to a final volume of 3 mL of establishment medium. Generally, try to ensure there are enough cells to achieve 25-50% confluency in the flask the cells are being seeded into.
27. As mentioned in Step 23, there are situations in which cells from a tissue explant culture should be passaged prior to approaching 50% confluency:
  a. Cells start proliferating in mounds outside of the tissue piece which could potentially prevent nutrients from reaching the centre of the mass, leading to cell death.
  b. Outgrowth from tissue pieces merge and prevent additional outgrowth.
  c. Outgrowth from tissue pieces reach the flask wall.
  d. In the above cases, where overall cell numbers are lower, we have opted to re-seed the cells into the same flask containing the original tissue explants. However, if the cells appear to be actively proliferating at a high rate, it may be advisable to transfer the detached cells into a new flask.
28. If tissue pieces remain attached to the original flask after subculturing, add 3 mL of establishment medium to the flask containing the tissue explants and place the flask back in the incubator to allow for potential further outgrowth and subculturing of cells from the primary tissue piece. **Additional Important Notes**:
  a. Many of the tissue pieces may exhibit initial cell migration but may not necessarily sustain productive cell outgrowth and proliferation.
  b. Tissue pieces which detach at any point should be removed from the tissue culture flask using a sterile transfer pipette and discarded. We have found that these tissue pieces do not reattach and thus do not produce cell lines.
  c. Some tissue pieces which remain adherent after initial treatment with trypsin solution will continue actively proliferating, while others will not. Maintain and monitor these tissue culture explants for chances to generate additional cell lines from same tissue piece; these can be used as a backup if cultures generated from earlier trypsinization attempts fail. Switch from establishment medium to early passage medium with flasks containing tissue explants after 2 months of initiation; flasks containing tissue explants should not be maintained for more than 4-6 months.
  d. Complete medium exchanges should be resumed after the first subculturing event.
29. Following the first passage of the cell culture, exchange half of the medium using establishment medium twice a week. Closely observe if these frequent partial medium changes aid or hinder cell outgrowth. Reduce the frequency of partial medium changes to once a week if cell growth slows. Once the cell culture reaches 90 – 95% confluency, subculture at a 1:2 ratio. The time to subsequent subculture varies greatly (can require up to 2 – 3 weeks).
30. Continue to monitor and subculture the successful cell cultures regularly. Growth dynamics and rates can change drastically in these early stages. Carefully observe the cells daily using phase-contrast microscopy to estimate the doubling rate of the cell culture and whether this rate is changing with successive passaging.
31. Once the cell cultures surpass passage 4, begin to maintain them in early passage medium and perform complete medium changes, as necessary. Continue subculturing the cells at a 1:2 ratio; it is recommended that cells cultivated at these early passages be used for cryopreservation (see *Cryopreservation* Section). It is advisable to expand the number of early passage cells to (1) increase the number of early passage cell lines that are cryopreserved, and (2) to supplement a duplicate flask with cell-conditioned medium from the same cell culture to determine whether cell-conditioned medium might support the growth of the cells.
32. After the cell line has stabilized (conformed to a predictable subculture schedule), usually around passage 15 – 20, culture conditions should be optimized (outlined in the next section).
33. As skin tissue consists of multiple cell types, cell lines should be further characterized using molecular markers that can be used to discern cell type. Marker selection should consider the diversity of cells present in the tissue being used to generate the culture and the species from which the cell line originated. We selected several molecular markers when characterizing the epithelial-like nature of the Xela DS2 and Xela VS2 cell lines that we established using this protocol [3].
34. Lastly, further characterization of ploidy and/or karyotype of the cell lines could be evaluated and monitored over the life of the cell line to assess for undesirable changes in the cell line.

## Important considerations for initiating and establishing frog cell lines

1. Material permitting, prepare multiple flasks of tissue culture explants from each tissue. Try multiple culture conditions but do so in a controlled manner to determine what conditions work the best at promoting initial cell outgrowth and proliferation.
2. Careful consideration should be given to medium formulation and multiple media formulations might need to be tested to achieve initial establishment of the primary cell culture and the continued cell growth required to generate a cell line. There are many medium parameters which can be manipulated and effect cell growth (positively or negatively), including osmolarity, types/concentrations of salts, types/concentrations of amino acids, and types/concentrations of sugars. If facing difficulty with establishing a cell culture because there is not enough outgrowth or the cells look “unhappy” (vacuolization, signs of apoptosis, loss of adherence), then consider altering these parameters. Below we describe the observations made and examples of the steps we took that helped to promote the generation of a *R. sylvatica* skin cell line:
  a. Observation: Initial cell outgrowth within the first week followed by vacuolization in cells.
    i. We optimized medium osmolarity by manipulating the proportion of L-15 medium mixed with cell culture water and found A_60_L-15 to facilitate greater cell outgrowth and longer cell survival. However, delayed cellular vacuolization was still observed.
    ii. We added β-mercaptoethanol to the establishment medium. β-mercaptoethanol is a common culture medium additive which can enhance cell survival and growth by reducing concentrations of harmful reactive oxygen species and aiding protein synthesis by increasing cell uptake of cystine [4]. This appeared to mitigate cellular vacuolization and we were able to perform an initial subculture of the cells from the tissue explant. We were able to subculture the cells a few more times, but ultimately the cultures failed to continue growing.
    iii. We supplemented the medium with cell-conditioned medium from a *Xenopus laevis* dorsal skin epithelial-like cell line (Xela DS2), in conjunction with β-mercaptoethanol. We found that the addition of Xela DS2 CCM supported the growth of *R. sylvatica* cells up to passage 10, where we were then able to grow the cells in Rasy complete medium (supplemented with CCM from the *R. sylvatica* skin cell line) or in the absence of any CCM, although the cell line required additional time to reach confluency in the absence of any CCM.
  b. Observation: Slowing of cell outgrowth.
    i. Perform partial medium exchanges instead of complete medium exchanges.
    ii. Supplement growth medium with cell-conditioned medium from the same, or similar cultures.
3. Every cell culture system is different and what is most important to successfully establish a continuous cell culture is constant monitoring to help determine what is “normal” for the culture and what manipulations promote growth.
4. Be prepared to be incredibly patient. Establishing a cell line can be a very slow process and can potentially take up to a year or more before the cell line is stable.

### Optimization of culture conditions for frog cell lines

1. It is necessary to determine the optimal growth parameters for cell cultures. We have described the first three parameters previously [3] and have included a fourth parameter, the addition of cell-conditioned medium, below.
  a. FBS supplementation – the growth of each cell line in response to varying percentages of serum supplementation should be experimentally determined to identify the optimal level and the lower limit of serum supplementation [3]. We have commonly found a range of FBS serum supplementations to be suitable for a cell line and have generally opted to select the higher serum supplementation level identified in the optimum range to ensure an excess of necessary growth factors. We have found a 15% FBS supplementation level to be suitable for a wide range of cell lines from different species and tissue types.
  b. Temperature – frogs are ectotherms, meaning that their body temperature varies directly with their surrounding environment. As such, the optimal growth temperature of frog cell cultures varies depending on the species and should be carefully considered when initiating a tissue culture explant and maintaining the cell line(s) generated from those tissue explants. We have successfully generated and maintained *X. laevis* frog skin cell lines at 26 °C and *R. sylvatica* skin cell lines at 18 – 20 °C.
  c. Seeding cell density – it is important to determine whether the initial cell number that is seeded affects cell growth. In some cases, aggressive subculturing (too frequently and/or at too low of a cell density) leads to loss of the cell line, presumably due to decreased cell-to-cell contact and/or loss of threshold levels of endogenous cell-secreted molecules into the cell culture medium.
  d. Addition of cell-conditioned medium (CCM) – CCM refers to medium collected from confluent flasks of cell lines (outlined in the *Collection of CCM* Section)*.* CCM contains numerous cellular proteins and metabolites released through secretory pathways, such as growth factors and hormones, which accumulate over time until the medium is renewed. The addition of CCM to newly prepared growth medium may improve cellular growth and aid in cell culture establishment. We have found that supplementing growth medium with 10 – 30% CCM can help in the establishment of *X. laevis* and *R. sylvatica* skin cell lines from tissue explants and/or is necessary to maintain some cell lines.
  e. **IMPORTANT:** When implementing these optimized parameters, it is important to test the new growth parameters over multiple subcultures and to keep a set of flasks maintained at the original growth parameters the cell line was established with.
2. **IMPORTANT:** Serum supplementation levels should remain consistent during maintenance of each cell line, once optimized, and serum lots should always be matched and further tested alongside a serum sample that is known to promote growth of the cell line. Always record the lot number of the serum used in establishing cell lines and/or maintaining cell lines.
  a. Serum type – we have found FBS to provide the necessary supplementation for the base medium. Attempts to use newborn calf serum or gamma-irradiated FBS failed – in both cases the cell lines would proliferate for a short while and then crash after three or more subculture efforts.
  b. Lot by lot variation – no two lots of FBS or any serum type from an animal origin are identical and differences in FBS lots can negatively impact cell growth. It is important to obtain a sample of a FBS lot prior to its purchase in bulk and test this FBS on the cell lines over multiple passages to ensure they continue to grow at the same rate. We generally buy FBS in bulk to minimize the need for lot testing. As with the serum type, some lots of FBS were incompatible with the cell lines and resulted in the loss of cell growth.

### General maintenance of frog skin cell lines

1. Once a cell line has been established (regular subculturing schedule, optimization of growth parameters completed), maintain cells in complete medium (does not contain antibiotics). **NOTE**: The rate of cell proliferation may increase over the first 15 – 20 passages of the cell line. This is likely a result of proliferative cells persisting while long-lived senescent cells die out. This is normal in our experience; however, the cells eventually settle into a “rhythm” after they are established, permitting accurate prediction of the date that they will need to be subcultured. Conversely, cell proliferation rate can decrease with high passage numbers as the cells become senescent.
2. Monitor cell lines regularly for changes in cell morphology, rate of growth, and/or signs of microbial contamination. We recommend taking images of the cells after every 5^th^ passage (e.g., passage 5, 10, 15…) to document cell morphology and recording dates of cell culture passage to estimate time between passages.
3. Upon reaching 90 – 95% confluency, cell lines should be subcultured in a biosafety cabinet:
  a. Aspirate the medium from the cell culture.
  b. Wash the cell culture with APBS by placing 2 – 3 mL of APBS or 4 – 5 mL of APBS into the 25 cm^2^ or 75 cm^2^ flask, respectively, using a serological pipette. Gently rock the APBS across the growth surface of the flask. **NOTE:** Different cell lines can have different strengths of attachment to the tissue culture vessel and time to cell detachment will vary. If cells are difficult to detach, try washing the cells with APBS (2 – 3 mL) two or three times to remove excess protein from the flask that may prevent timely detachment of cells.
  c. Aspirate the APBS.
  d. Quickly add amphibian-adjusted trypsin solution for cell detachment (1 mL for a 25 cm^2^ flask, 2 mL for a 75 cm^2^ flask) using a serological pipette and rock the solution over the growth surface. **NOTE:** We have generally used amphibian-adjusted trypsin solutions for cell detachment. Consider experimenting with different detachment methods such as a cell lifter or other salt-based cell dissociation buffers, depending on the tolerance of the cell line to these detachment methods. Some cell types and/or cell lines are quite sensitive to certain treatments, whether physical or chemical, so it is important to know which detachment methods are suitable for use with a particular cell line or for a particular downstream application.
  e. Cap the flask and remove from the biosafety cabinet. Monitor the flask for detachment of cells from the tissue culture vessel. Once most cells have detached (greater than 95%), return the flask to the biosafety cabinet.
  f. Add an equal volume of complete medium to the flask and gently pipette to dissociate cell clumps.
  g. Subculture the cells at the pre-determined ratio. We generally subculture cell lines at 1:2 or 1:4 and is dependent on the cell line. **IMPORTANT:** It is critical to test, in parallel, different subculture ratios for a cell line in parallel with the 1:2 subculture for an extended period (at least 4 – 5 passages) as some cell lines will die out if split at too high a ratio.
  h. Add fresh complete medium to the subcultured cells and return the flask to the incubator.
4. Duration between passages will vary based on the cell line and incubation temperature. In our experience, it can be every 3 – 4 days or up to every 14 days. **IMPORTANT:** For cell lines that are passed every 7 or 14 days, a full or partial medium exchange may be required in between subculture dates.
5. Plating efficiency of each cell line should be empirically determined with respect to the chosen detachment method. If the detachment method is altered, plating efficiency must be redetermined.

### Collection of CCM

There are many benefits of saving the CCM of a cell line, such as improving cellular growth and aiding in cell culture establishment. Here, we describe the method used for collecting CCM and recommendations for its use.

1. CCM collection can occur simultaneously with subculture of the cell line.
2. Working in a biosafety cabinet, use a sterile serological pipette to collect the medium from the confluent culture and transfer the CCM to a sterile conical tube. The collected CCM can be processed immediately or stored at 4 °C for later processing as a batch.
3. Centrifuge the CCM at 1,000 × *g* for 10 min to pellet large particulates or cellular debris.
4. In the biosafety cabinet, filter the CCM into a sterile vessel using a 0.22 µm filter (bottle top or syringe filter). Some CCM can be viscous and thus care should be taken to not apply high levels of pressure that could result in breakage of the filter. **IMPORTANT:** Do not disturb the cell pellet – transfer only the medium for filtration.
5. The sterile CCM can be stored at 4 °C and used for several weeks. However, it may be preferable to use fresh CCM as the stability of the cell-produced factors is unknown.
6. Not all cell lines require CCM; the requirement for the presence and percentage of CCM required for each individual cell line should be determined empirically.

### Cryopreservation

It is important to cryopreserve continuous cell cultures at early (passage 5 – 10) and mid passages (passage 10 – 50), if compatible with the cell culture or cell line. It is necessary to empirically determine the optimal cryopreservation medium and cryopreservation agent (type and percentage) and determine whether the cell line is tolerant of cryopreservation, and if so, for how long. Detailed below is the method which has been most successful in our experience working with *X. laevis and R. sylvatica* skin cell lines*.*

1. Before harvesting cells, prepare a mixture of 80% complete medium and 20% dimethyl sulfoxide (DMSO). Chill on ice and protect the mixture from direct light.
2. Pre-label an appropriate number of cryopreservation tubes (cryovials) with the name and passage number of the cell line to be preserved, the date of cryopreservation, and the preservation medium. **IMPORTANT:** Update records for storage of cryopreserved cell lines.
3. Transfer the harvested cells to a conical tube and close the lid on the tube.
4. Remove the tube from the biosafety cabinet and centrifuge the cell suspension at 300 × *g* for 10 min to pellet the cells.
5. Return the conical tube to the biosafety cabinet and carefully aspirate the supernatant.
6. Resuspend the pellet in a known volume of complete medium and place the tube on ice.
7. Remove a small volume of the cell suspension (∼100 µL) and combine it with an equal volume of 0.4% Trypan Blue dye, gently pipetting to mix. Load the cell-dye mixture onto a hemocytometer and perform a cell count to determine the cell density of the suspension.
8. Dilute the cell suspension in complete medium to 2 × 10^6^ cells/mL. Aliquot 0.5 mL of the cell suspension to each cryovial and place on ice.
9. Working with one tube of cells at a time, add 0.5 mL of the pre-prepared ice-cold 80% complete medium / 20% DMSO solution to the cryovial containing the 500 µL of cell suspension. This will yield 1 mL of 1 × 10^6^ cells/mL in 90% complete medium / 10% DMSO (v/v).
10. Immediately cap the tube, gently invert 5 – 10 times, and place on ice. Work through the remaining tubes quickly.
11. Next, remove the icebox from the biosafety cabinet and transfer the tubes to a freezing container, such as a Mr. Frosty.
12. Immediately transfer the freezing container holding the cryovials of cells to a -80 °C freezer for overnight storage (12 – 16 h).
13. The following day, transfer the cryovials to a liquid nitrogen tank for long term storage. **IMPORTANT:** Use appropriate personal protective equipment (insulated gloves, safety goggles, lab coat) when handling liquid nitrogen and ensure to record the location of each cryovial in a laboratory inventory. We have found long-term storage of frog cell lines at -80 °C to be unsuitable; storage in liquid nitrogen is required.

### Resurrection of cryopreserved frog cell lines

1. To prepare for thawing cryopreserved cells, first:
  a. Warm a water bath to ∼30 °C.
  b. In the biosafety cabinet, add 9 mL of cold (4 °C) medium to a 15 mL conical tube and place it on ice. The medium placed in the tube should be the growth medium of the cell line. We use cold medium in this step to keep cell metabolism low until all DMSO can be removed from the thawed cells.
  c. Place the bottle of growth medium on ice to keep it cool.
  d. Pre-label the flask the cells are to be placed in. We recommend listing the name of the cell line, passage number, the date the cells were cryopreserved, date the cells were resurrected, and the initials of the individual initiating the culture.
  e. Place a beaker of warm water from the water bath inside the biosafety cabinet.
2. Retrieve the cryovial from the liquid nitrogen tank and place the vial in the biosafety cabinet. **IMPORTANT:** Open the cap slightly to relieve any pressure inside the vial in the event liquid nitrogen entered the vial during storage.
3. Holding the top of the cryovial just under the lid, place the bottom of the cryovial in the warm water bath and continuously swirl the cryovial in the warm water to ensure even warming of the contents. **IMPORTANT:** Do not allow the cryovial to fall into or be submerged in the water. It is very important that the top of the cryovial stay out of the water to prevent potential contamination of the cells.
4. As soon as the liquid has thawed sufficiently (some ice will still be present) to allow for transfer of the contents to the prepared 15 mL conical tube, it is important to perform the next steps quickly as DMSO (as well as other cryoprotectant agents) is toxic to cells. **IMPORTANT:** Continuous swirling of the tube is needed to ensure the contents of the tube stay cold and keep cell metabolism low.
5. Remove the cryotube from the water bath and spray the tube generously with 70% ethanol. Wipe away excess ethanol.
6. Use a 1000 µL micropipette to transfer the thawed medium/cells from the cryovial to the 15 mL conical tube. Close the tube and mix by gentle inversion. This rapidly dilutes the DMSO.
7. Centrifuge the conical tube at 230 × *g* for 10 min at room temperature. **IMPORTANT:** The cells may be fragile – centrifuge at low speeds.
8. After centrifugation, return the tube to a biosafety cabinet and aspirate the medium. **IMPORTANT:** Do not disturb the cell pellet.
9. Using a serological pipette, transfer the cooled growth medium to the 15 mL conical tube and gently pipette to resuspend the cell pellet. We use 3 – 4 mL of growth medium if the cells are to be placed in a 25 cm^2^ plug seal flask or 10 mL of growth medium if the cells are to be placed in a 75 cm^2^ plug seal flask. We opted to use cooled growth medium to minimize additional temperature shock of the cells and this appeared to enhance recovery of our cell lines. Once in the incubator, the medium will gradually rise to the temperature set in the incubator. However, cells could be directly resuspended in growth medium at room temperature or pre-warmed to the incubation temperature. As with other steps, optimization is often required to find ideal conditions for each cell line.
10. Transfer the cell suspension into the appropriate plug seal tissue culture treated flask. Rock the flask to ensure even dispersal of the cells across the growth face of the flask.
11. Remove the flask from the biosafety cabinet and place the flask in an incubator overnight set to the optimal growth temperature.
12. The next day, observe resurrected cells with phase-contrast microscopy to verify cell adhesion to the flask. If there are many floating cells, aspirate the medium from the flask in a biosafety cabinet and replace the medium using growth medium that has been warmed to the optimal growth temperature to avoid a temperature shock to the cells.
13. Replace the flask in the incubator and continue daily monitoring to watch for recovery of the cells.

## Method validation

The Xela DS2 and Xela VS2 cell lines established in Bui-Marinos et al. 2020 [3] using the methodology described herein have become valuable tools to elucidate antiviral responses present in frog skin epithelial cells and uncover how pathogens may subvert such responses [3,5,6].

## Declaration of Competing Interest

The authors declare that they have no known competing financial interests or personal relationships that could have appeared to influence the work reported in this paper.
